# A New Framework for Job Shop Integrated Scheduling and Vehicle Path Planning Problem

**DOI:** 10.3390/s26020543

**Published:** 2026-01-13

**Authors:** Ruiqi Li, Jianlin Mao, Xing Wu, Wenna Zhou, Chengze Qian, Haoshuang Du

**Affiliations:** 1 Faculty of Mechanical and Electrical Engineering, Kunming University of Science and Technology, Kunming 650032, China; 20231103005@stu.kust.edu.cn (R.L.);; 2 Faculty of Information Engineering and Automation, Kunming University of Science and Technology, Kunming 650032, China

**Keywords:** job shop scheduling problem with limited transportation, multi-robot path planning, reinforcement learning, PBS algorithm

## Abstract

With the development of manufacturing industry, traditional fixed process processing methods cannot adapt to the changes in workshop operations and the demand for small batches and multiple orders. Therefore, it is necessary to introduce multiple robots to provide a more flexible production mode. Currently, some Job Shop Scheduling Problems with Transportation (JSP-T) only consider job scheduling and vehicle task allocation, and does not focus on the problem of collision free paths between vehicles. This article proposes a novel solution framework that integrates workshop scheduling, material handling robot task allocation, and conflict free path planning between robots. With the goal of minimizing the maximum completion time (Makespan) that includes handling, this paper first establishes an extended JSP-T problem model that integrates handling time and robot paths, and provides the corresponding workshop layout map. Secondly, in the scheduling layer, an improved Deep Q-Network (DQN) method is used for dynamic scheduling to generate a feasible and optimal machining scheduling scheme. Subsequently, considering the robot’s position information, the task sequence is assigned to the robot path execution layer. Finally, at the path execution layer, the Priority Based Search (PBS) algorithm is applied to solve conflict free paths for the handling robot. The optimized solution for obtaining the maximum completion time of all jobs under the condition of conflict free path handling. The experimental results show that compared with algorithms such as PPO, the scheduling algorithm proposed in this paper has improved performance by 9.7% in Makespan, and the PBS algorithm can obtain optimized paths for multiple handling robots under conflict free conditions. The framework can handle scheduling, task allocation, and conflict-free path planning in a unified optimization process, which can adapt well to job changes and then flexible manufacturing.

## 1. Introduction

With the advancement of intelligent manufacturing and workshop digitization, Job-Shop Scheduling (JSP) has become increasingly uncertain and spatiotemporal coupled due to factors such as multi-resource coupling [1], complex processes, and constrained logistics. The goal of JSP is to determine processing sequences and resource allocation for multiple machines handling a series of operations. This problem has been proven NP-hard [2,3], and in real-world scenarios, it often faces challenges like random order arrivals, equipment status fluctuations, and delivery constraints, evolving into a dynamic problem requiring higher algorithmic adaptability and real-time performance. With the development of artificial intelligence, data-driven approaches such as deep learning (Yuan and Li) [4] and machine learning (Das et al.) [5] have garnered significant attention from researchers. A crucial step in applying reinforcement learning to JSP is its formulation as a Markov Decision Process (MDP) [6], involving key definitions of actions, states, and rewards. However, previous studies often relied on custom formulations based on domain knowledge of JSP, employing manual engineering and specific workloads, leading to inconsistent experimental results. As Liu, Chang, and Tseng [7] have noted regarding state space, some studies define states as matrices composed of customer order characteristics and system features (such as machine quantity and average completion time), though they fail to specify relationships between different entities. Other research employs disjunction graphs to represent original states and utilizes graph neural networks (GNN) for feature extraction (Zhang [8]). The primary advantage lies in its ability to handle instances of varying scales without requiring additional training. However, GNN performance may degrade significantly for complex problems, with computational costs potentially increasing substantially (Wu et al. [9]), as increased neighborhood node numbers may propagate noise information (Zhou et al. [10]). Since conventional JSPs disregard transportation routes, we need to introduce a multi-robot path problem to address workpiece transportation in shop floor scheduling.

Beyond processing stages, material handling constitutes a critical component in modern workshops. Traditional scheduling research focusing on “material handling” often oversimplifies operations by treating transportation as a fixed time or capacity constraint, without explicitly modeling paths, congestion, or collision avoidance. This leads to solutions that lack inherent conflict-free implementability. It is worth noting that the integration of manufacturing scheduling and AGV transportation is not limited to Job Shop scenarios but is also extensively studied in Flexible Flow Shop (FSP) domains. Both fields face similar challenges in resource coordination and collision avoidance. For instance, recent research by (Wang [11]) proposed a mixed-integer linear programming model for efficient flexible flow shop scheduling with automatic guided vehicle consideration. However, distinct from FSP which typically follows a unidirectional or stage-sequential flow, JSP involves more complex, multi-directional routing constraints, imposing higher demands on path planning flexibility. To bridge this gap, recent studies have integrated transportation resources into scheduling models, with the landmark work being the Flexible Job Shop Scheduling Problem with Limited Automated Guided Vehicle(AGV) Transportation (FJSP-LAT) [12]. This approach coordinates machinery and limited AGV fleets in flexible workshops, establishing an optimization model that minimizes maximum completion time, total energy consumption, and delivery penalty through multi-objective evolutionary algorithms. Experimental data reveals diminishing marginal returns from increasing AGV numbers, providing quantitative evidence for resource allocation. However, most existing FJSP-LAT literature neglects explicit path conflict resolution for AGVs, typically adopting a modeling assumption that “ignores path conflicts and collisions between AGVs” (key assumptions listed in the model section) [13]. This disconnect persists between the planning (scheduling) and execution (path planning) layers, requiring additional multi-robot path planning and collision mitigation during implementation phases. In summary, a critical synthesis of the literature reveals a significant dichotomy: purely scheduling-focused research tends to oversimplify logistics into static time lags, failing to account for dynamic congestion; meanwhile, routing-focused studies often lack the global foresight required for production efficiency. Consequently, most existing hybrid approaches struggle to balance computational tractability with the topological realism needed for actual industrial deployment. This highlights the urgent need for a framework that inherently unifies task dispatching with deadlock-free path verification. Recent trends in hybrid optimization-learning frameworks emphasize the need to balance computational speed with execution feasibility. For industrial-level implementation, a key requirement is ensuring that algorithmic outputs strictly adhere to physical constraints (e.g., collision avoidance and kinematic limits), which purely data-driven methods often struggle to guarantee probabilistically. By integrating the rigorous constraint-handling capability of PBS with the adaptive decision-making of RL, our proposed framework aligns with these industrial standards, offering a solution that is both efficient in calculation and reliable in physical execution.

The proposed model effectively bridges the gap where Job Shop Scheduling with transportation typically neglects collision-free paths among multiple robots, while specific multi-robot path planning algorithms overlook task-level dispatching. First, in problem formulation: We propose a variant of the Job-Scheduling Problem (JSP) that explicitly incorporates transportation time and path accessibility, enabling dispatch plans with direct implementability without adding additional optimization objectives. Second, in modeling: An integrated mixed-integer/Discrete-Time Network (MIDN) model is developed to unify decision-making processes for “transportation decisions”, AGV assignment, and collision-free path selection within a single timeline, while ensuring no space occupation conflicts or head-on collisions through vertex-edge conflict constraints. Third, in methodology: A collaborative solution process integrating “RL scheduling and PBS path planning” is established. The scheduling layer generates high-quality initial Gantt charts under optional machine sets and exclusion constraints, while the path layer creates collision-free transport plans for workstation nodes. Real-time transportation duration then enables closed-loop adjustments to scheduling details. Fourth, in experimentation: Our approach demonstrates effectiveness through classical cases including FT, LA, SWV, and YN. Most instances achieve or exceed strong baselines in scheduling quality. Taking FT06 as an example, the integrated Gantt chart after path fusion perfectly aligns with both time dimensions and AGV routes, achieving zero collisions. This validates the practicality and scalability of the “workshop scheduling + path planning” framework.

## 2. Problem Description and Modeling

### 2.1. Problem Description

This paper aims to consider the job shop scheduling (JSP) and automated guided vehicle (AGV) path planning problems simultaneously, with the optimization objective of minimizing the maximum completion time (makespan) in the overall manufacturing system. Specifically, the problem is described as follows:

(a) A manufacturing workshop system comprises multiple processing machines, each equipped with independent input and output nodes.
(b) Workpieces within the facility must undergo sequential processing through multiple stages, with each step requiring completion exclusively on designated machines without interruption.
(c) AGVs (Automated Guided Vehicles) transport workpieces between processes, with each vehicle limited to handling a single piece at any given time.

The workshop’s processing tasks are defined as a set of processes, each with a fixed processing time. Additionally, the internal logistics routes within the workshop are modeled as an undirected connected graph. The node set represents critical positions such as machine entry points, exit points, initial and final workpiece locations, while the edge set indicates the paths that AGVs can traverse.

Each AGV is assigned a fixed initial position and operates in time units defined as time steps, during which it can either remain stationary or move to adjacent nodes. The system must specify the AGV’s transportation routes and durations for each process, along with determining the processing start times for each operation. The joint optimization problem proposed in this paper requires simultaneous decision-making across multiple processes:

(a) The processing sequence and time scheduling of the workpiece on each machine;
(b) Task allocation and path planning of AGV to avoid path conflict and node occupancy conflict among multiple AGVs.

The constraints include but are not limited to:

(a) Each workpiece must be processed in the predetermined order;
(b) Each machine can process at most one process at any time;
(c) Each AGV can transport at most one workpiece at any time;
(d) At any time, each path node or edge can only be occupied by one AGV at most.

The optimization objective is defined as minimizing the overall maximum completion time while considering AGV transportation time under the aforementioned constraints. This problem holds significant theoretical and practical implications, effectively enhancing production-logistics coordination efficiency. It prevents scheduling conflicts and congestion during AGV operations, ensuring efficient and orderly execution of production tasks.

**Figure 1:**
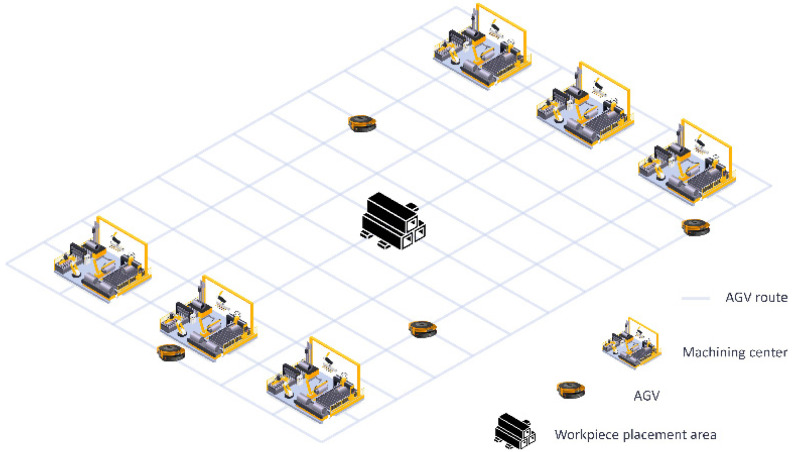
Example of the combination of JSP and MAPF problems.

Figure 1 illustrates an application combining JSP and Multi-Agent Path Finding (MAPF) problems. Each machining machine M serves as both the entry point (waiting area) and exit point for workpieces, with multiple robots freely performing transportation tasks across the facility. Upon completion of a machining process, the AGV transports the workpiece to the next machine in the workflow until all tasks are completed, after which the workpiece is returned to the central warehouse in the map.

**Figure 2:**
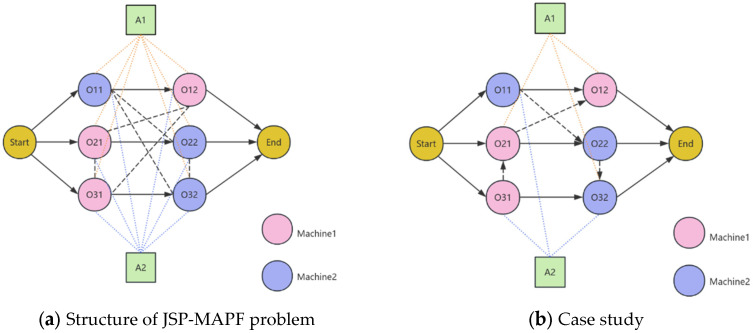
Structure of JSP-MAPF problem and Case study.

To visually demonstrate the JSP-MAPF problem involving process sequencing and AGV allocation, Figure 2a illustrates the problem structure with two processing machines, three workpieces, and each workpiece requiring two processes along with two AGVs. The solid lines represent the actual process sequence, while the dashed lines indicate the potential sequencing order and transport tasks.Here, Oij denotes the j-th process of the i-th workpiece, where blocks represent AGVs, solid lines indicate processing sequences between workpieces, black dashed closed lines show potential sequence relationships before processing on the same machine, and colored dashed lines indicate potential AGV assignments for each process (where AGVs are dispatched to subsequent machines after completing current processes). Figure 2b presents a solution case: Machine 1 follows processing sequences O31, O21, O12, etc., with Robot A1 handling the transportation of processes O21, O32, O12, and so on.

### 2.2. Mathematical Model

In order to accurately describe the problem, this section gives a mathematical description of our problem. Table 1 shows the definition of symbols, Table 2 shows the decision variable.

As the Table 3 shows, Objective function (1) aims to minimize the maximum completion time for machining and AGV transportation. Constraints (2)–(4) represent machine processing constraints. Specifically, Constraints (2)–(3) enforce sequence constraints: each process must be assigned to exactly one designated machine, with the completion time equal to the start time plus the processing duration. Constraint (4) implements the machine overlap constraint: any two processes assigned to the same machine must be processed sequentially through the mutual exclusion of M constraints. Constraints (5)–(6) govern task assignment constraints: when adjacent processes occur on different machines, the subsequent process must not start earlier than the predecessor’s completion time plus the workpiece transportation time, and each task must be executed by exactly one AGV. Constraints (7)–(9) enforce path planning constraints. Constraint (7) ensures path feasibility by maintaining the spatiotemporal continuity of the AGV. Constraints (8) and (9) address multi-robot conflict constraints: they restrict the maximum occupancy of a node to one vehicle (vertex collision constraint) and prohibit opposing travel on the same route (edge collision constraint).

## 3. Solution of JSP Considering Transportation Time and Path

Building upon the model definition, this chapter unfolds in the sequence of “scheduling layer → task allocation layer → multi-AGV path planning layer”: First, it presents the MDP design and training strategies for the reinforcement learning scheduler; then, automatically generates and assigns transportation tasks through Gantt chart preprocessing; subsequently implements PBS (Priority-Based Search) for multi-AGV non-conflict path planning on workshop topology; finally, feeds the arrival times of paths back into the timeline to complete the sequential integration of processing and transportation.

**Figure 3:**
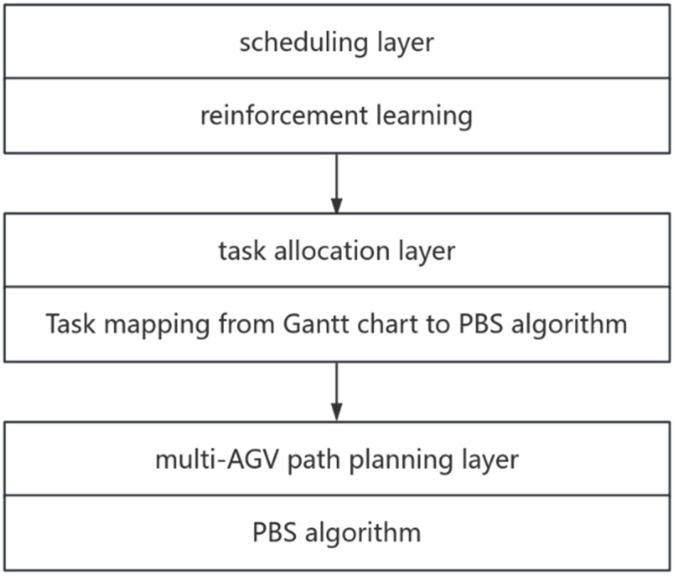
Solution Framework.

In the path consideration FJSP problem, the optimization goal is to minimize the transportation time and processing time, and the combinatorial optimization of this combinatorial optimization problem is carried out at three levels: machine processing scheduling layer, robot task allocation layer and path planning layer, respectively.As illustrated in Figure 3 below.

### 3.1. Introduction to Machining Scheduling Framework

The JSP uses the scheduling framework from the paper [6]. The scheduling layer framework of this study aims to achieve efficient decision-making and execution of workshop scheduling tasks to minimize completion time. Specifically, we adopt the D3QPN algorithm (incorporating Dueling Double DQN with Prioritized Experience Replay) to effectively mitigate Q-value overestimation and enhance sample efficiency within the discrete scheduling action space.The scheduling layer framework includes the following key components:

1. State representation: The scheduling problem is described in a structured manner through the partitioning diagram. In this diagram, nodes represent the processing procedures to be processed, and edges represent the constraints before and after processing as well as machine usage constraints. This structured representation can effectively capture the timing relationship of the procedures and resource constraints.
2. Feature extraction module: The feature extraction module uses the Attention mechanism to extract features from the disjunction graph, so as to effectively capture the key information in the graph structure and the correlation features between processes.
3. Action selection module: The general dispatching rules (such as FIFO, LIFO, SPT, LPT, etc.) are adopted as the action space. According to the results of graph feature extraction, the most suitable dispatching rules for the current state are dynamically selected to balance the efficiency and interpretability of decision-making.
4. Scheduling execution and status update: According to the selected scheduling rules, the task scheduling of corresponding number of steps is performed, and the workshop environment and task status information are updated to provide real-time status feedback for the next decision.
5. Feedback and iterative updates: Based on the effect of scheduling execution, reward feedback is calculated and the strategy is continuously updated through reinforcement learning to improve the overall scheduling performance.

**Figure 4:**
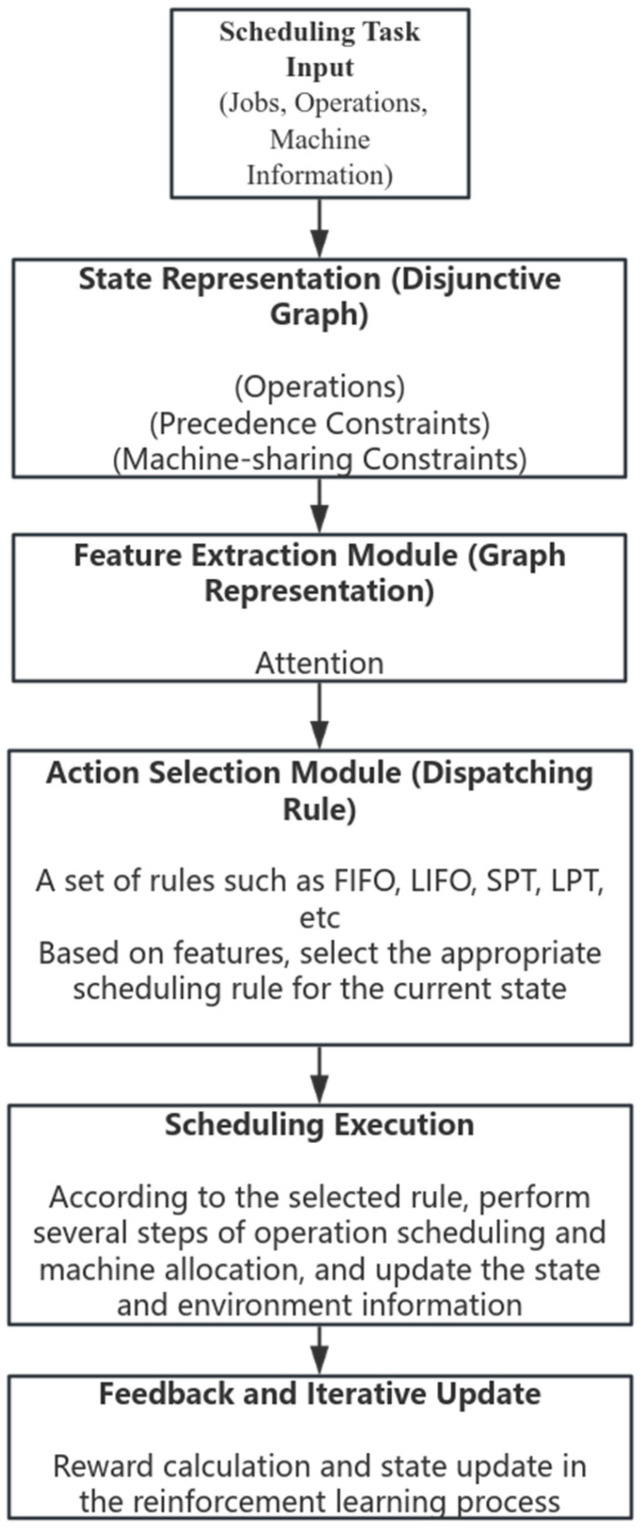
Framework for solving JSP by reinforcement learning.

The scheduling layer framework is shown in Figure 4 below. It has the characteristics of clear structure, easy to implement and interpret, and can effectively adapt to complex workshop scheduling and AGV transportation collaborative optimization tasks.

#### Action Selection Improvements

The scheduling actions of JSP in reinforcement learning are crucial for addressing scheduling challenges. In the action selection module of the aforementioned framework, at time t, decisions must be made regarding processing actions for multiple workpieces. The original method included: (1) First-In-First-Out (FIFO); (2) Last-In-First-Out (LIFO); (3) Most Remaining Operations (MOR); (4) Least Remaining Operations (LOR); (5) Longest Processing Time (LPT); (6) Shortest Processing Time (SPT); (7) Longest Total Processing Time (LTPT); (8) Shortest Total Processing Time (STPT). While these actions are designed based on process characteristics, scheduling fundamentally involves sequencing and machine allocation. Since none of the existing actions specifically address machine optimization, we propose two new strategies: Most Idle First (MIF) and Least Idle First (LIF), incorporating them into the original eight action set.

**Figure 5:**
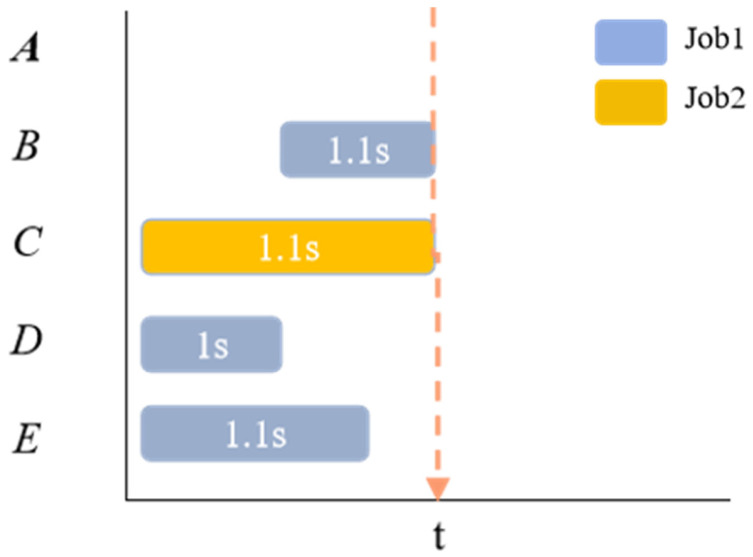
Action selection.

As shown in Figure 5 below, there are five processing machines numbered A–E. At time t, MIF prioritizes the process that can be processed on machine A for arrangement, while LIF prioritizes the process that can be processed on machine C for arrangement.

### 3.2. Robot Task Assignment

Upon the completion of machine processing scheduling, transportation tasks are generated and assigned to the executable AGVs. Each task corresponds to the movement of a workpiece for job i after completing operation j. Formally, this is represented as a Transport Request ($T_{req}$) triplet:

$$T_{req}(i,j)=\langle L_{start},L_{end},t_{ready}\rangle$$

where $L_{start}$ denotes the docking node $L_{ij}$ of the current machine, $L_{end}$ denotes the docking node $L_{i,j+1}$ of the target machine for the subsequent operation, and $t_{ready}$ corresponds to the operation completion time $C_{ij}$.

Task Allocation Strategy: The scheduler distributes the set of requests to the robot set V. The allocation process determines the decision variable $z_{ik}$ to ensure that each task is assigned to exactly one robot, satisfying the assignment constraint:

$$\sum_{k\in V}z_{ik}=1,\;\forall i\in J$$

This mechanism ensures that all generated transportation requests are covered by the available fleet.

Idle Priority Principle: To optimize transport efficiency, an “Idle Priority Principle” is employed. When a task $T_{req}(i,j)$ becomes available at $t_{ready}$, the system identifies the subset of idle robots, $V_{idle}\subseteq V$. The assignment algorithm selects the optimal robot $k^{*}$ by minimizing the travel cost to the starting node:

$$k^{*}=\underset{k\in V_{idle}}{\arg\;\min}\{Cost(path_{k},L_{start})\}$$

The task is assigned to the selected robot $k^{*}$ (setting $z_{ik^{*}}=1$), thereby minimizing the waiting time before the transportation begins.

### 3.3. Preprocessing of Scheduling Results Combined with Path Planning

Traditional Gantt charts developed for scheduling problems often neglect transportation time. When incorporating this factor, two critical issues emerge: (1) The continuous influx of different processes onto a single machine creates transportation challenges; (2) Within the same workpiece, the immediate transfer to the next machine after completing a previous process fails to account for transportation duration.

According to the above problems, we use the buffer to solve the first problem and the buffer to preprocess the Gantt chart to solve the second problem.

This section details the preprocessing workflow for integrating path planning-generated transit windows with actual transportation durations into the scheduling timeline. For each material handling task, the start time of the next process is determined by taking the maximum value between the “machine availability” and “transport arrival time”. This approach ensures that the final maximum completion time Cmax incorporates material handling duration while maintaining strict temporal alignment between Gantt charts and the original routing path.

#### 3.3.1. Setting of Handling Buffer Zone

**Figure 6:**
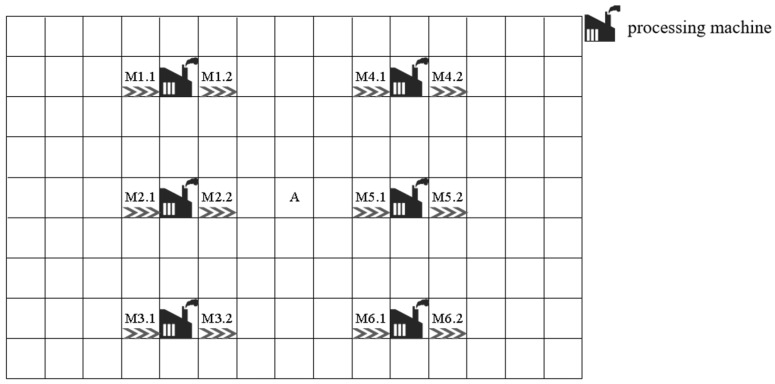
JSP map instance.

To enhance the implementability of the scheduling algorithm, this study incorporates a distinct workshop spatial architecture during modeling, as illustrated in Figure 6. The functions of the symbols are shown in Table 4.The system comprises machining units, input/output ports, and buffer zones, where X.1 denotes the unified entry point for workpieces and X.2 represents the centralized exit point. Each machining unit is equipped with both input and output buffer zones to temporarily store workpieces awaiting processing or completed items requiring transportation. This design addresses the unrealistic assumption in traditional scheduling models that “different operations on the same machine can seamlessly transition without interruption.” In real-world production environments, workpiece transportation and storage inevitably introduce additional delays. The buffer zones serve as intermediaries, allowing workpieces to temporarily reside in these zones after processing until transportation resources or target machines become available for transfer.

Specifically, during the material handling process, AGVs first enter the global buffer through Entrance (Position 1), where they are transported to corresponding processing units via conveyor belts or robotic arms. Upon completion of processing, workpieces are transferred to the output buffer of their respective units, awaiting subsequent transportation. All workpieces ultimately exit the system through Exit (Position 2). This mechanism not only considers processing and transportation in the temporal dimension but also ensures rational workpiece flow in the spatial dimension, effectively preventing scheduling infeasibility caused by resource conflicts or transportation delays.

#### 3.3.2. Workpiece Buffer Treatment

**Figure 7:**
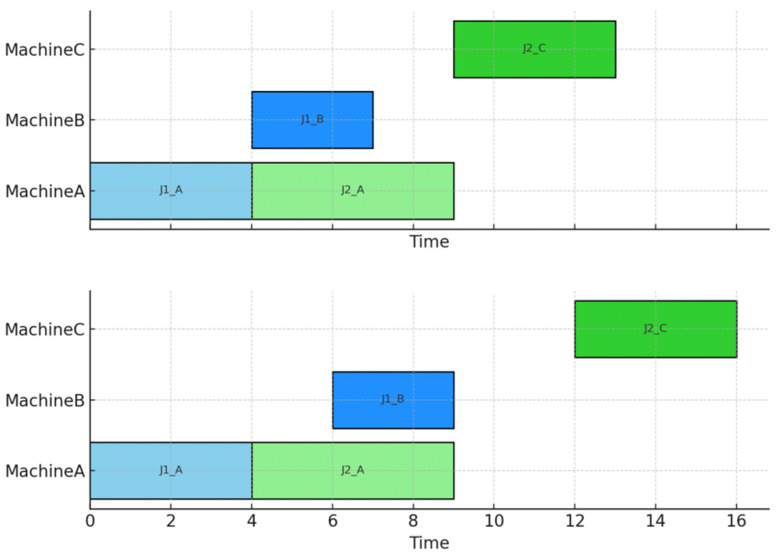
Gantt preprocessing.

Figure 7 demonstrates the comparison between traditional scheduling results and the preprocessing method proposed in this study. The upper chart shows a conventional Gantt chart that ignores transportation processes, where workpieces are immediately processed on the next machine after completing the previous step. However, in real-world production environments, workpieces require transportation to reach target equipment, making such scheduling unrealistic in practice. The lower chart presents the preprocessed Gantt chart generated by our method, which explicitly incorporates transportation time and resource constraints between processes. For instance, after MachineA completes processing of workpiece J1, its path planned by the PBS algorithm requires 2 time units to reach Machine B, causing subsequent processing tasks to be delayed accordingly. Similarly, the transportation process between MachineA and MachineC for workpiece J2 takes 3 time units, pushing subsequent processing tasks to time point 12. This demonstrates that our method effectively addresses the issue of unreasonable process sequencing caused by traditional scheduling ignoring transportation, generating scheduling solutions that better align with practical execution conditions. This preprocessing mechanism lays the foundation for integrated optimization of path planning and scheduling in subsequent stages.

#### 3.3.3. Task Mapping from Gantt Chart to PBS Algorithm

Traditional approaches only assign processing sequences and machine assignments at the scheduling layer without explicitly outputting transportation tasks. This results in the path layer struggling to inherit scheduling outcomes and align with the timeline, often leading to “disconnection between transportation and machining” during execution phases. To address this, we need to automatically and systematically extract transportation tasks from the obtained Gantt chart as input for the PBS algorithm, ensuring precise spatiotemporal correspondence between path planning and scheduling.

**Figure 8:**
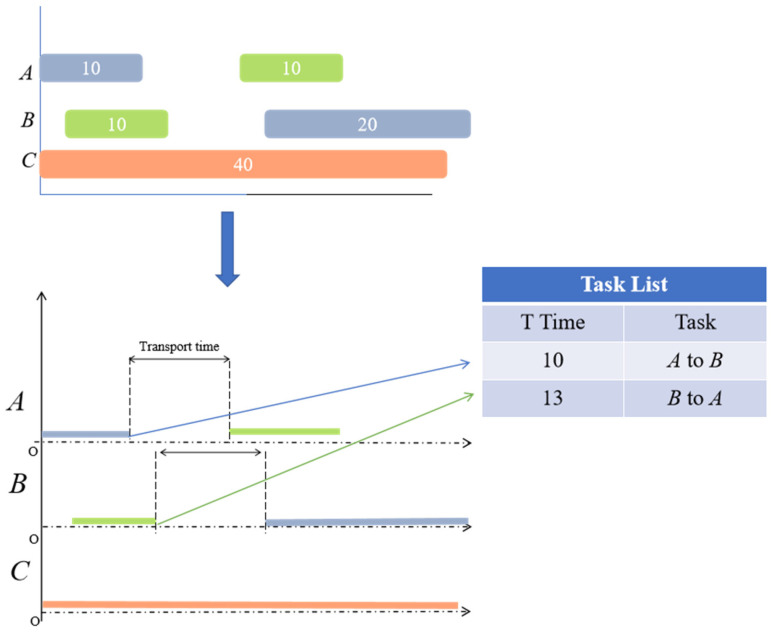
Gantt chart task extraction.

Through preprocessing, we ensure all processes can be successfully transferred. Using “adjacent processes across different machines” as the trigger condition, we verify each process’s completion status before determining if it is the final step. The system extracts critical data from the Gantt chart—specifically task response time T and task transition X to X—to generate task lists for PBS system processing. As illustrated below, this demonstrates a simplified workflow for task extraction.As illustrated in Figure 8.

### 3.4. Path Planning Layer PBS Algorithm

Based on the extracted task list, we utilize the PBS (Priority-Based Search) algorithm to determine the underlying path for material handling within a given workshop map. This algorithm integrates the principles of prioritized planning and conflict-driven search, ensuring solvability while significantly improving computational efficiency. The PBS framework divides the search process into two hierarchical levels:

High-Level Search (PT Priority Tree): Maintains a priority graph. Initially, no priority constraints exist, and each robot independently plans the optimal path. The system checks for vertex conflicts or edge conflicts in the path set. If a conflict is detected (overlapping paths between robots), the Priority-Based Search (PBS) expands two child nodes in the priority tree: Node 1: robot (i.e., priority). Node 2: robot. This gradually constructs a partial priority relationship, and the system performs depth-first traversal of the priority tree until finding a conflict-free path set.

Low-Level Search (LLS): Monocular Path Planning: Given a priority relationship, the LLS algorithm plans paths for robots one by one. During path planning, high-priority robots maintain fixed routes while low-priority robots must avoid these paths (treated as dynamic obstacles). The system employs a Space-Time A* algorithm to identify collision-free paths.

Note: Description of Symbols and Functions To ensure consistency with the mathematical model in Section 2.2, the notations in Algorithm 1 are defined as follows:

V: The set of AGVs $\left\{v_{1},\ldots ,v_{K}\right\}$, corresponding to Table 1.

$T_{req}$: The collection of transport requests generated in Section 3.2.

$SpaceTimeA^{*}(k,\ldots )$: The low-level pathfinding function that calculates the optimal path for AGV k while treating higher-priority agents as dynamic obstacles.

priorities: A set of ordering constraints ($e.g.,k_{high}≻k_{low}$) used to resolve conflicts; AGV $k_{low}$ must yield to $k_{high}$.

FindFirstConflict(…): A function that validates whether the path set satisfies the Vertex Collision Constraint (Equation (8)) and Edge Collision Constraint (Equation (9)).

Stack: A data structure executing a Depth-First Search (DFS) on the priority tree.
**Algorithm 1:** PBS for Factory Transport$Input:\;\mathrm{Workshop}\;\mathrm{Topology}\;G(N,E);\;\mathrm{Set}\;\mathrm{of}\;\mathrm{AGVs}\;V=\{v_{1},\ldots ,v_{K}\};\;\mathrm{Transport}\;\mathrm{Requests}\;T_{req}\;;$$Output:\;\mathrm{Collision}-\mathrm{free}\;\mathrm{path}\;\mathrm{set}\;\Pi =\{path_{k}\;|\;k\in V\};$     
Root.priorities←∅
     
for each agent k∈V do
             
$path_{k}\leftarrow SpaceTimeA^{*}(k,\emptyset )$
             
$if\;path_{k}\;\mathrm{is}\;\mathrm{not}\;\mathrm{found}\;then\;return\;Failure$
     
$Root.paths\leftarrow \{path_{1},\ldots ,path_{K}\}$
     
$Stack.push\left(Root\right)$
     
while Stack is not empty do
         
Node←Stack.pop()
         
Conflict←FindFirstConflict(Node.paths)
         
if Conflict is None then return Node.paths
         
$\left(k_{a},k_{b})\leftarrow GetConflictingAgents(Conflict\right)$
         
$for(k_{high},k_{low})\;\mathrm{in}\;\{(k_{a},k_{b}),(k_{b},k_{a})\}\;do$
            
Child←Node.copy()
            
$Child.priorities.add(k_{high}≻k_{low})$
            
$Child.paths[k_{low}]\leftarrow SpaceTimeA^{*}(k_{low},Child.priorities)$
            
$if\;Child.paths[k_{low}]\;\mathrm{is}\;\mathrm{found}\;then$
              
Stack.push(Child)
     
return Failure


## 4. Result

### 4.1. Strengthen the Verification of Scheduling Algorithm

To verify the effectiveness of this algorithm at the scheduling layer, programming was conducted on Python 3.11, with the operating environment being Windows 11 operating system, 2.5 GHz, and 8 GB RAM. The selected comparative algorithm is GA [14], A2C [15], PPO [16], DQN [17], Rainbow [18], D3QPN [6].We utilized widely recognized classic JSP benchmark instances, including the Ft series [19], La series [20], Swv series [21], and Yn series [22].”

Table 5 presents scheduling results across nine classic JSP benchmark instances (Ft, La, Swv, Yn series), comparing heuristic/evolutionary algorithms (GA) with various deep reinforcement learning methods (A2C, PPO, DQN, Rainbow, D3QPN). The evaluation metric is Makespan, where lower values indicate better scheduling quality. Our method outperformed the current best solutions in three instances (La31, Swv06, Yn1) and tied for the top in five others (La01, La06, La11, La21, Swv01), with only Ft06 showing a 1-unit time lag behind the optimal solution. Using average row values (AVG) as reference, our method achieved an average relative improvement of 9.7% across all instances. Compared to the best solutions, it demonstrated an average relative difference of −0.41%, reflecting performance levels comparable to or slightly superior to current optimal solutions.

To validate the effectiveness of the proposed MIF and LIF actions across different scheduling environments, an ablation study was conducted on FT06 (small-scale), La31 (medium-scale), and Yn1 (large-scale) instances, as the Table 6 shows. The results indicate a positive correlation between the performance gain of the proposed strategy and the problem scale. In the small-scale FT06 instance, the baseline algorithm already reached the performance ceiling (Makespan maintained at 59) due to the limited solution space, showing no additional gain but confirming stability. As the problem complexity increased to the medium-scale La31 (30 × 10), the MIF and LIF actions began to identify optimization opportunities missed by standard rules, achieving a refinement in Makespan from 1775 to 1770, which marks the inflection point of the strategy’s effectiveness. The most significant improvement was observed in the large-scale Yn1 (20 × 20) instance, where the Makespan decreased substantially from 1098 to 1044 (approximately 5%). This demonstrates that in high-dimensional environments with intense resource competition and fragmented idle times, explicitly optimizing machine idleness effectively overcomes local optima, confirming the superior scalability and robustness of the proposed action space design for complex scheduling problems.

### 4.2. Scheduling Problem Considering Transportation Path

To evaluate the performance of the proposed framework in realistic production environments, this section conducts integrated experiments combining machine scheduling and AGV path planning. We selected the classic small-scale instance FT06 (6 × 6) and the large-scale instance LA11 (20 × 5) as test beds.

Standard JSP benchmarks and multi-robot systems lack a unified time dimension; therefore, adopting a direct 1:1 conversion ratio is empirically unreasonable. To address this discrepancy, we implement a 1:10 temporal scaling strategy. Specifically, machine processing times are amplified by 10 to align with the finer time granularity required for AGV path planning.

#### 4.2.1. Small-Scale Instance Verification

We first validated the method using the FT06 instance, configuring the system with 4 AGVs to handle material transport tasks. The theoretical optimal makespan for this instance (without routing constraints) is 59.

**Figure 9:**
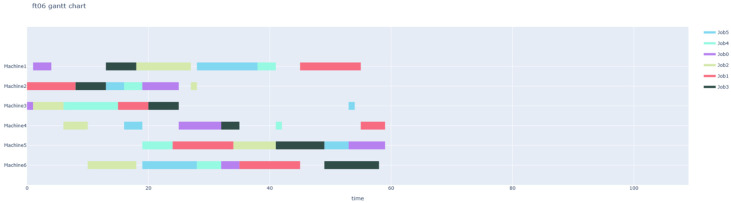
FT06 original Gantt chart.

**Figure 10:**
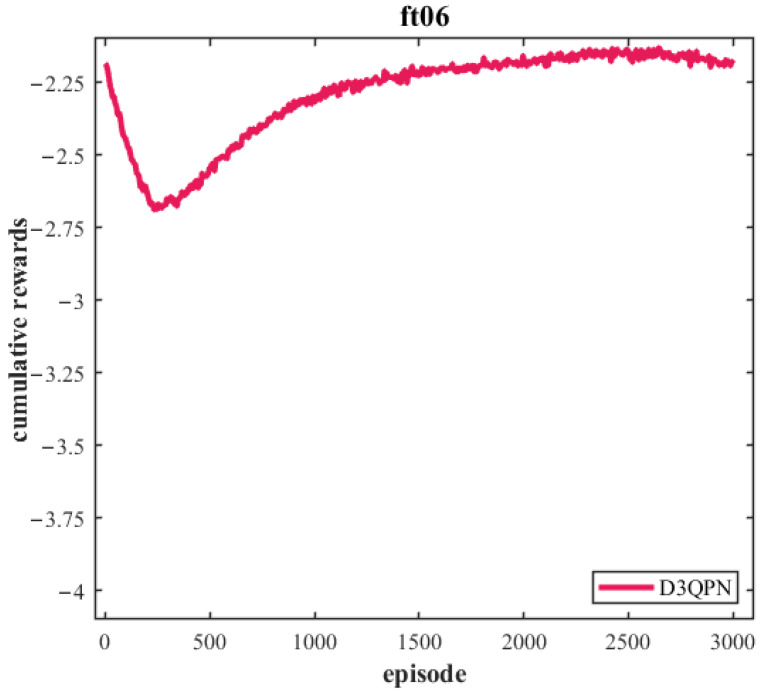
Training Curves.

Figure 9 shows the initial Gantt chart generated by the reinforcement learning scheduler. While the sequence is optimized, it ignores the spatial transfer time between machines. Figure 10 shows its training curve.

**Figure 11:**
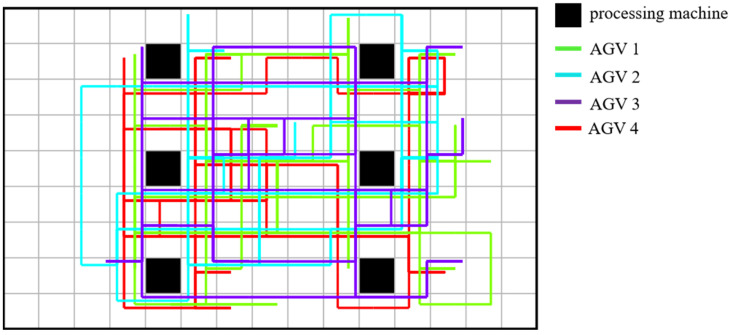
Robot path diagram.

**Figure 12:**
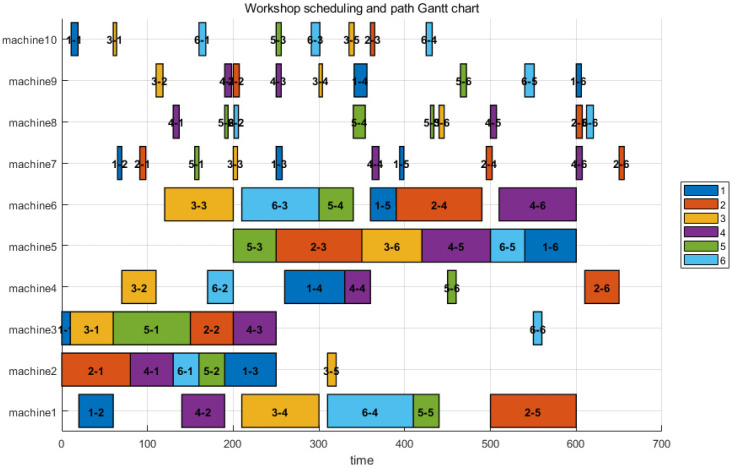
**FT06** Scheduling and Path Gantt Chart.

Based on the extracted task list, the PBS algorithm generated conflict-free paths for the 4 AGVs on the grid map, as shown in Figure 11. These paths account for dynamic obstacle avoidance and congestion. The final integrated Gantt chart (Figure 12) yields a makespan of 654 under the 1:10 scaling setting. When normalized back to the original scale, this corresponds to 65.4. Compared to the theoretical optimal of 59, the difference of 6.4 units (approximately 10.8%) represents the inevitable time cost introduced by logistics transportation. This confirms that a fleet of 4 AGVs effectively supports the production rhythm with reasonable logistics overhead.

#### 4.2.2. Large-Scale Robustness Analysis

**Figure 13:**
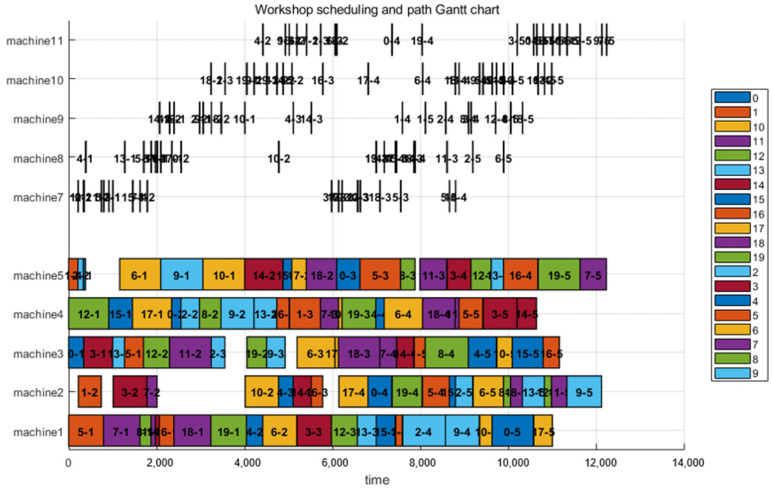
LA11 Scheduling and Path Gantt Chart.

To verify the scalability and robustness of the algorithm under complex conditions, we introduced the LA11 instance (20 jobs, 5 machines).The results are presented in Figure 13. Given the increased workload, the fleet size was slightly adjusted to 5 AGVs. The theoretical optimal value for LA11 is 1222.Applying the same 1:10 scaling strategy, the theoretical baseline becomes 12,220. The experimental results for the joint scheduling of LA11 are as follows: The final integrated makespan is 12,835. The absolute difference between the experimental result (12,835) and the scaled baseline (12,220) is 615. Remarkably, the relative time increase caused by transportation is only 5.03%

Compared to the FT06 instance (4 AGVs, 10.8% increase), the LA11 instance (5 AGVs, 5.03% increase) demonstrates a significantly lower relative logistics cost. This indicates that even with a minimal increase in fleet size (from 4 to 5), the proposed algorithm can effectively coordinate the parallel processing capabilities of machines and the AGV fleet to “absorb” transportation delays, demonstrating excellent global optimization capabilities in complex scenarios.

#### 4.2.3. Summary of Joint Optimization Results

Table 7 summarizes the experimental results. The data confirms that the proposed method ensures zero-collision transportation while maintaining high scheduling efficiency across different scales and fleet configurations.

## 5. Conclusions

This paper presents an integrated solution framework structured into three layers—scheduling, task allocation, and multi-robot path planning—to address the JSP-T problem, where transport robots are traditionally not considered in conflict-free path generation. The framework aims to minimize the makespan, explicitly incorporating transportation time. In the scheduling layer, a reinforcement learning algorithm is employed, and its action design is refined to produce high-quality processing-sequence plans. Experiments on FT, LA, SWV, YN, and other benchmark instances show that the proposed method improves the makespan by 9.7% compared with other reinforcement-learning approaches such as PPO. In the task allocation layer, the scheduling plan is mapped to task times and locations, and transportation tasks are assigned to robots, enabling an effective integration of the JSP and MAPF formulations. In the multi-robot path planning layer, the PBS algorithm receives tasks, workshop layouts, and transportation-resource information to generate conflict-free paths efficiently and reliably for multiple robots. Overall, the framework provides a coherent optimization pipeline from scheduling to execution, enhancing adaptability to varying shop-floor operations and supporting flexible manufacturing environments. Finally, by minimizing high-energy machinery idle time and preventing redundant AGV movements, the proposed framework contributes to energy conservation, directly aligning with the social imperative of sustainable ‘Green Manufacturing.

It is important to clarify that the proposed framework operates primarily as a two-stage sequential optimization strategy rather than a fully dynamic, real-time closed-loop system. Specifically, the mechanism relies on generating an initial high-quality schedule which is then refined via a one-time feedback adjustment based on the collision-free paths generated by the PBS algorithm. While this “Generate-then-Refine” approach effectively resolves spatiotemporal conflicts and ensures implementability, it has limitations. The global optimality of the final solution is partially dependent on the quality of the initial schedule, and the current offline nature of the framework limits its ability to handle continuous, real-time disturbances during execution. Future work will focus on extending this mechanism into a fully iterative loop to further enhance robustness in dynamic environments.

## Figures and Tables

**Table 1:** Meaning of Symbolic Variables.

Symbol	Description
J	Set of jobs, i∈J={1,2,…,n}
$O_{ij}$	The J-th operation (process) of job i
M	Set of machines, $m\in M=\{1,2,\ldots ,m_{total}\}$
V	Set of AGVs (robots), k∈V={1,2,…,K}
$P_{ij}$	Processing time of operation $O_{ij}$ on its assigned machine
$L_{ij}$	The docking node (position) of the machine assigned to $O_{ij}$
G(N,E)	Workshop topology graph; N is the set of nodes, E is the set of edges 3
$M_{\infty }$	A sufficiently large positive constant (Big-M)

**Table 2:** Decision variable.

Symbol	Description
$S_{ij}$	Start time of operation $O_{ij}$ on the machine
$C_{ij}$	Completion time of operation $O_{ij}$ on the machine
$x_{ijm}$	Binary variable: 1 if $O_{ij}$ is processed on machine m, 0 otherwise
$y_{iji^{\prime }j^{\prime }}$	Binary variable: 1 if $O_{ij}$ precedes $O_{i^{\prime }j^{\prime }}$ on the same machine, 0 otherwise
$z_{ik}$	Binary variable: 1 if the transportation of job i is assigned to AGV k, 0 otherwise
$path_{k}(\tau )$	The node position of AGV k at discrete time step τ
$T_{trans}(i,j,j+1)$	Actual travel duration for job i from $O_{ij}$ to $O_{i,j+1}$

**Table 3:** Mixed integer linear programming model.

$\min C_{\max}=\max_{i,j}(C_{ij})$	(1)
$C_{ij}=S_{ij}+P_{ij}$	(2)
$\sum_{m\in M}x_{ijm}=1$	(3)
$S_{i,j+1}\geq C_{ij}+T_{trans}$	(4)
$S_{i^{\prime }j^{\prime }}\geq C_{ij}-M_{\infty }(1-y_{iji^{\prime }j^{\prime }})$	(5)
$\sum_{k\in V}z_{ik}=1$	(6)
$path_{k}(\tau +1)\in \{path_{k}(\tau )\}\cup Adj(path_{k}(\tau ))$	(7)
$path_{k}(\tau )\neq path_{k^{\prime }}(\tau ),\;\forall k\neq k^{\prime }$	(8)
$\left(path_{k}(\tau ),path_{k}(\tau +1))\neq (path_{k^{\prime }}(\tau +1),path_{k^{\prime }}(\tau )\right)$	(9)

**Table 4:** Map information.

Node Type	Symbol	Functional Description
Workshop site	A	Initial/end position
Processing entry	Mn.1	Processing task starting point
Export processing	Mn.2	Completion point of processing tasks

**Table 5:** Benchmark Comparison.

Instance	Size	GA	A2C	PPO	DQN	Rainbow	D3QPN	Ours
FT 06	6 × 6	58	69	67	65	63	59	59
La01	10 × 5	738	830	828	785	935	718	718
La06	15 × 5	982	1043	1021	984	1066	926	926
La11	20 × 5	1330	1225	1331	1283	1480	1222	1222
La21	15 × 10	1502	1334	1345	1347	1494	1286	1286
La31	30 × 10	2436	2075	2047	1958	1846	1775	1770
Swv01	20 × 10	2319	1979	1986	1962	2061	1813	1813
Swc06	20 × 15	2960	2369	2354	2311	2333	2175	2170
Yn1	20 × 20	1496	1250	1132	1109	1110	1098	1044

**Table 6:** Comparison of Ablation Experiments.

Instance	Size	8 Action	+MIF	+LIF	+Both
FT 06	6 × 6	59	59	59	59
La31	30 × 10	1775	1772	1772	1770
Yn1	20 × 20	1098	1098	1076	1044

**Table 7:** Performance Analysis.

Instance	Size	AGV Num	Optimal	Scaled Base	Ours	Transport Cost
FT 06	6 × 6	4	59	590	654	10.8%
LA11	20 × 5	5	1222	12,220	12,835	5.03%

## Data Availability

Data Availability Statement: The raw data used in this study are openly available as third-party data from Zeng et al. [6] at https://github.com/Yunhui1998/Gymjsp (accessed on 3 December 2025). The processed data generated from these raw data and used in the analyses of this article are available from the corresponding author upon reasonable request.
